# Antiparasitic Effects of Sulfated Polysaccharides from Marine Hydrobionts

**DOI:** 10.3390/md19110637

**Published:** 2021-11-12

**Authors:** Natalya N. Besednova, Tatyana S. Zaporozhets, Boris G. Andryukov, Sergey P. Kryzhanovsky, Svetlana P. Ermakova, Tatyana A. Kuznetsova, Anastasia N. Voronova, Mikhail Y. Shchelkanov

**Affiliations:** 1 G.P. Somov Research Institute of Epidemiology and Microbiology, Federal Service for Surveillance on Consumer Rights Protection and Human Wellbeing, 690087 Vladivostok, Russia; niiem_vl@mail.ru (T.S.Z.); andrukov_bg@mail.ru (B.G.A.); takuznets@mail.ru (T.A.K.); avoronova92@gmail.com (A.N.V.); adorob@mail.ru (M.Y.S.); 2 School of Biomedicine, Far Eastern Federal University (FEFU), 690091 Vladivostok, Russia; 3 Medical Association of the Far Eastern Branch of the Russian Academy of Sciences, 690022 Vladivostok, Russia; priemmodvoran@mail.ru; 4 G.B. Elyakov Pacific Institute of Bioorganic Chemistry, Far Eastern Branch of the Russian Academy of Sciences, 690022 Vladivostok, Russia; svetlana_ermakova@hotmail.com; 5 National Scientific Center of Marine Biology, Far Eastern Branch of the Russian Academy of Sciences, 690041 Vladivostok, Russia; 6 Federal Scientific Center of the East Asia Terrestrial Biodiversity, Far Eastern Branch of the Russian Academy of Sciences, 690022 Vladivostok, Russia

**Keywords:** sulfated polysaccharides, marine hydrobionts, antiparasitic activity, protozoa, malaria, leishmaniasis, trypanosomiasis, schistosomiasis, cryptosporidiosis, trichomoniasis

## Abstract

This review presents materials characterizing sulfated polysaccharides (SPS) of marine hydrobionts (algae and invertebrates) as potential means for the prevention and treatment of protozoa and helminthiasis. The authors have summarized the literature on the pathogenetic targets of protozoa on the host cells and on the antiparasitic potential of polysaccharides from red, brown and green algae as well as certain marine invertebrates. Information about the mechanisms of action of these unique compounds in diseases caused by protozoa has also been summarized. SPS is distinguished by high antiparasitic activity, good solubility and an almost complete absence of toxicity. In the long term, this allows for the consideration of these compounds as effective and attractive candidates on which to base drugs, biologically active food additives and functional food products with antiparasitic activity.

## 1. Introduction

Protozoal infections (protozoans) caused by parasites belonging to unicellular protozoa remain among the most common human diseases. Protozoa cause severe and often fatal diseases in humans as well as domestic and game animals. About 50 species of protozoa are known to cause disease in humans [1].

Parasites are a very diverse group of organisms that have developed various strategies for infecting hosts and living off them, parasitizing in various organs and tissues including the blood, intestines, central nervous system, liver, lungs, etc. These pathogenic microorganisms can be transmitted to humans through the alimentary route, through arthropod vectors, and sexually. There are three main classes of human pathogenic parasites: protozoa, helminths, and ectoparasites. 

Among protozoal infections, malaria [2], leishmaniasis [3,4], trypanosomiasis [5] and intestinal protozoa [6] are of the most significant medical and social importance. Many of the drugs currently used to treat protozoal infections do not meet modern requirements due to toxicity, gradual loss of effectiveness, and the emergence of drug resistance in parasites. Thus, there is an urgent need for new drugs.

Sources of new drugs for the treatment of protozoal infections can be natural products, synthetic molecules, or existing drugs with an extended spectrum of indications. A combination of new methods, including genetic modification of pathogens, bioimaging, molecular docking [7], and robotics has led to the standardization of high-throughput screening platforms for drug discovery [8]. For the treatment of protozoal infections, new effective agents are needed which do not cause at all or cause a minimum of side effects and to which protozoa will not develop resistance in a short time.

Extracts of many seaweeds have pronounced antiparasitic properties [9,10,11,12], and have therefore been studied on a wide range of human parasites, such as protozoa (*Plasmodium* spp., *Leishmania* spp., *Trichomonas* spp., etc.) [10,13,14] and helminths (*Neobenedenia* spp.) [15]. Algae extracts can inhibit the binding of parasites to target cells or have a direct toxic effect on protozoa. Thus, R. Moo-Puc et al. [13] showed direct antiprotozoal activity of organic extracts obtained from several algae against the trophozoites of *Trichomonas vaginalis*. At the same time, 44% of the studied seaweed varieties exhibited high or moderate antitrichomonal activity. M.S. Coutinho [16] reported a strong larvicidal effect of extracts of two algae, *Fucus vesiculosus* and *Ulva lactuca* (sea lettuce) against the larval stages of the *Aedes aegypti* mosquito.

However, the authors of this review do not set the goal of a detailed analysis of the results of studying the biological effectiveness of extracts of marine aquatic organisms, since these contain many biologically active substances with different chemical natures. Further in-depth research is required to determine the active component of each. In addition, the chemical composition of the extracts of aquatic organisms depends on the type of algae, the region of extraction, the method of extraction, extragents, and many other factors.

In this regard, sulfated polysaccharides (SPS)—sulfated homo- and heteropolysaccharides which are agonists of receptors of innate and adaptive immunity cells—are of great interest in this regard. They are characterized by low toxicity and the absence of side effects (except in cases of allergic-type reactions when using heparin with an admixture of highly sulfated polysaccharides [15,16]). These biopolymers have various biological actions including antiviral and antibacterial, anti-inflammatory, and immunomodulatory activity [17,18,19]. SPS encompasses a diverse group of anionic polymers found in various marine organisms from macroalgae to mammals, but have not been found in terrestrial plants [19].

It is known that the most common heteropolysaccharides in the human body are glycosaminoglycans (GAGs), negatively charged long unbranched polymeric polysaccharides consisting of repeating units of disaccharides [20]. The binding of glycosamines to various ligands leads to post-translational modifications that ensure cell migration, proliferation, differentiation, etc. Among GAGs, heparin/heparan sulfates are of particular interest. These substances are present in basement membranes, in the extracellular matrix, and on the surface of cells as part of membranes. They can specifically interact with macromolecules of the extracellular matrix (fibronectin, laminin), enzymes, and a wide class of heparin-binding molecules (growth factors, chemokines) [21]. GAG mimetics, including heparin/heparan sulfates, bind to other macromolecules and interfere with signal transduction pathways in cells, which provides a wide range of biological effects. The natural mimetics of heparan sulfates are the sulfated polysaccharides of seaweed. Fucoidans, carrageenans, and ulvans, isolated from brown, red and green algae, respectively, can mimic the action of endogenous factors and regulate the functions of macroorganism systems through key receptors of cells and enzymes. In this regard, SPS can bind to various receptors on the host cell’s surface and compete with viruses, bacteria, and parasites for glycoprotein receptors [20,21].

An essential aspect of the action of SPS is their antiadhesive activity, which largely explains their antibacterial [22], antiviral [23], and as will be shown below, antiparasitic activity.

Fucoidans, SPS derived from brown algae, are the most commonly used in experiments. This is because fucoidans are an essential structural component of algae and account for up to 30% of their dry weight. Furthermore, the pharmacological safety of the polysaccharide is confirmed by absence of toxicity to humans even when consumed in amounts up to 3 g per day [24]. In addition, these biopolymers have a systemic effect, as evidenced by their detection in blood plasma [24].

There are many reports in the literature in which the results of experiments are presented indicating the effectiveness of using SPS from marine aquatic organisms (in most cases, marine macroalgae) as an antiprotozoal agent. In this regard, the purpose of this work is to generalize the available data on the interaction of these compounds with protozoan pathogens and assess the prospects for using them as a basis to create a new generation of antiparasitic drugs.

## 2. Malaria

Malaria is a life-threatening transmission parasitic disease that is transmitted to humans through the bites of infected female *Anopheles* mosquitoes [25]. This infection continues to be relevant for global health [26]; in 2019, 229 million people fell ill with malaria worldwide, and 409,000 people died. Most malaria cases and deaths, 94%, are reported in the African continent [27]. The most vulnerable contingent for this disease is children under five years of age [28].

The etiological agents of malaria are parasites of the genus *Plasmodium*, whose life cycle occurs in the body of two hosts: a human (intermediate host) and female *Anopheles* mosquitoes (final host). To date, more than 200 Plasmodium species have been officially described, each of which infects a certain range of hosts [29]. In humans, malaria is caused by five species of *Plasmodium*: *P. falciparum* (tropical malaria agent), *P. vivax* (the causative agent of three-day malaria), *P. malariae*, *P. ovale* (the causative agent of malaria oval), and *P. knowlesi*. The first four species are pathogenic to humans, while *P. knowlesi* causes infection in primates and causes zoonotic malaria in Southeast Asia [29]. The most dangerous are *P. falciparum* (mainly distributed in Africa) and *P. vivax* (distributed in the American continent).

All *Plasmodium* species possess high genetic plasticity, allowing parasites to adapt to changes in environmental parameters and rapidly develop resistance to antimalarial therapeutic agents [29]. When an infected female mosquito bites a person, malaria plasmodia at the sporozoite stage enter the bloodstream with insect saliva [26]. Within 10–30 min, they move freely with blood flow and then settle in the liver cells. Active tissue schizonts divide after 6–15 days with the formation of many tissue merozoites, which enter the blood after 1–6 weeks when the infected liver cells are destroyed. The merozoites then penetrate into the erythrocytes, after which the second cycle of erythrocytic schizogony begins.

Penetration of merozoites into erythrocytes is carried out in several stages: (a) initial contact and slight deformation of erythrocytes with the participation of surface antigens of merozoites; (b) severe deformation of erythrocytes with the participation of microneme proteins, rhoptry proteins and the actin–myosin motor of the parasite; (c) opening of pores between the parasite and erythrocytes; (d) internalization [30].

The attachment of merozoites to the membrane of erythrocytes and their invagination into membranes occurs due to the presence of specific receptors on their surface. In the erythrocyte, the merozoites go through four stages of development: i—stage of the ring (trophozoite); ii—stage of amoeboid schizont; iii—stage of morula (fragmentation); iv—formation of erythrocytic merozoites. The erythrocyte membrane is destroyed, and merozoites and their metabolic products are released into the plasma. At this time, the patient begins to suffer an attack of malaria (fever, anemia, and splenomegaly and, in tropical malaria, organ damage). Some of the blood merozoites again penetrate into the erythrocytes and repeat the entire cycle of erythrocytic schizogony. The duration of the erythrocytic schizogony phase is 72 h in *P. malariae* and 48 h in other plasmodia species [31].

Since erythrocytes cannot recycle the parasite and present antigens, the early stages of the erythrocyte ring remain invisible to the immune system until the later stages, trophozoites and schizonts, which modify the erythrocyte membrane in accordance with their needs for membrane transport [32,33]. Thus, the erythrocyte (clinical) stage of schizogony begins [34].

Some of the merozoites turns into gametocytes (immature germ cells). This process is called gametocytogeny. *P. falciparum* gametocytes develop in the deeply located vessels of internal organs. After maturation over a period of 12 days they appear in the peripheral blood, where they can remain viable anywhere from several days to up to 6 weeks. Gametocytes of other plasmodium species develop in peripheral vessels within 2–3 days and, after maturation, die after a few hours.

The most well-characterized is the surface protein of the parasite merozoite-1 (MSP-1), which is of great importance for the successful invasion of erythrocytes [35,36]. It is the largest, and covers the surface of the parasite. After binding to the erythrocyte, it reorients itself so that the apical end of the parasite is aligned with the erythrocyte membrane. This reorientation involves the apical membrane antigen, a transmembrane protein localized at the apical end of the merozoite and binding erythrocytes. The peripheral surface proteins of merozoites, MSP3, MSP6, MSPDBL1, MSPDBL2, and MSP7, bind directly to MSP1 independently of each other to form multiple forms of the MSP1 complex on the parasite’s surface. These complexes perform overlapping functions and directly interact with human erythrocytes [35].

Apicomplexan-type parasites, including *Plasmodium* spp., contain three unusual secretory organelles (microneme, rhoptries, and dense granules) required to infect new host cells [37]. Micronemes are located in the apical third of the plasmodium. These organelles secrete several proteins, including the apical membrane protein-1 of *P. falciparum* (PfAMA-1) and proteins of the EBA (erythrocyte binding antigen) family, which bind to receptors on the surface of red blood cells and facilitate the entry of plasmodium into them. It has been suggested that micronemes act in concert with roptria. While micronemes initiate erythrocyte binding, rhoptries secrete proteins to create a PVM, or parasitophorous vacuole membrane, in which the parasite can survive and reproduce [38,39].

Erythrocytes infected with mature forms of plasmodia bind to endothelial cells in capillaries by sequestration, which allows the parasite to multiply and evade destruction in the spleen [40]. Infected erythrocytes can adhere to uninfected ones, forming rosettes that can form clots by binding to other red blood cells mediated by platelets, which can lead to occlusion of the circulatory bed [41].

A significant problem is the ability of the parasite to develop resistance to antimalarial drugs such as chloroquine and sulfadoxine–pyrimethamine. Resistance also develops to artemisinin, part of the malaria elimination program [42,43]. This alarming situation requires the search for and development of new antimalarial agents with an original mechanism of action.

In developing antimalarial drugs, several approaches can be used, ranging from modifying existing agents to developing new agents that act against new targets [44]. Currently, most licensed antimalarial drugs target the intra-erythrocytic stage of the parasite. However, the invasion of merozoites—a pivotal moment in the development of parasites—has been shown by the studies of many authors to have great potential for affecting the surface proteins of plasmodia. Therefore, it is possible to use agents that inhibit the invasion of merozoites in combination with drugs aimed at destroying intraerythrocytic parasites [42,43,45,46,47].

The invasion of merozoites involves multiple ligand–receptor interactions and multiple pathways of parasitic invasion in host cells [46], which can be blocked by SPS and heparin-like molecules. The surface antigens of the parasite play a vital role in the process of *Plasmodium* invasion.

Studies have shown that SPS, in particular heparin (acidic sulfur-containing GAG) and heparin-like polysaccharides, have an antimalarial effect [47]. Heparin inhibits cytoadhesion and thus reduces parasitemia. On the apical surface of merozoites, various molecules are expressed that interact with receptors on the surface of erythrocytes. Heparin targets a large number of molecules on the parasite’s surface that mediate its invasion of red blood cells. In this regard, the authors believe that the risk of developing heparin resistance is very low because, apparently, many molecules at once will not be able to quickly acquire resistance to the polysaccharide. [48].

Heparin binds to proteins of the DBL and RBL families of the parasite; in its presence the interaction between the apical end of merozoites and the surface of erythrocytes is inhibited, and invasion does not occur. In this case, localization is limited only by the apical surface of the parasite, and the polysaccharide has only a slight effect on erythrocytes. A. Leitgeb et al. found that heparin equally inhibits both sialic acid-dependent and sialic acid-independent invasion of *P. falciparum* merozoites [49]. In addition, in the presence of heparin, deformation of the erythrocyte membrane is suppressed. However, heparin was discontinued due to the induction of severe bleeding in patients [45,49].

As an alternative, sevuparin was proposed, a heparin derivative with low anticoagulant activity and high anti-adhesive properties, which destroyed the rosettes (50% at a dose of 250 μg/mL). Sevuparin inhibits the cytoadhesion of plasmodia to epithelial cells, suppresses the invasion of merozoites into erythrocytes, and removes blockage of microcirculation, which alleviates life-threatening symptoms in children [50,51].

Studies carried out on the model of heparin and merozoites of *P. falciparum* have provided an understanding of the mechanism by which other SPS and similar molecules exert antimalarial effects.

**Figure 1:**
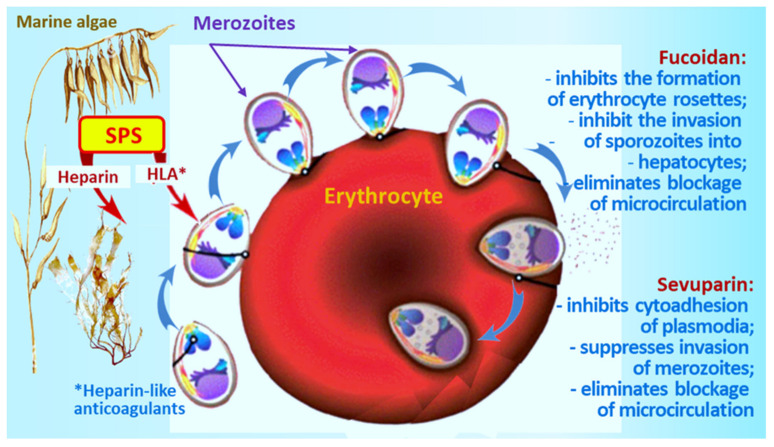
SPS from marine hydrobionts, in particular heparin, heparin-like polysaccharides, and fucoidans as well as the drug sevuparin developed on the same basis, all have antimalarial effects.

SPS from marine plants and invertebrates, which have molecular structures similar to heparin, also exhibited an anticoagulant effect [52,53]. These compounds are an exciting and promising alternative to heparin for antimalarial therapy and contain structures similar to pRBC-binding GAGs [54,55]. For quite a long time [56], the inhibitory effect of fucoidan (SPS of brown alga) on the formation of erythrocyte rosettes by plasmodia has been known; this activity extends to both wild and laboratory strains of the parasite. It has been shown that fucoidans inhibit the invasion of sporozoites into hepatocytes as well as the reinvasion of merozoites into erythrocytes (Figure 1).

Finally, in 2009, Chen et al. [57] presented the results of a study on the antimalarial effect of three fractions of fucoidan from the brown alga *Undaria pinnatifida*.

Experiments were carried out in vitro on *P. falciparum* culture and in vivo on mice infected with *P. berghei*. It was shown that all three fractions of the polysaccharide at a dose of 5 μg/mL were able to significantly inhibit the invasion of merozoites into erythrocytes, and the IC_50_ was similar for both chloroquine-sensitive *P. falciparum* 3D7 strain and K1 strain resistant to this drug. The most active was the fraction with the highest sulfate content. The survival rate of mice in the group of animals infected with *P. berghei* and receiving fucoidan (100 mg/kg) was 37.12 ± 0.37%, while among mice of the control group receiving chloroquine it was (5 mg/kg)—94.37 ± 0.94%. The protective effect of fucoidan was significantly lower in this case, but compared with the intact control (mice without treatment), the effect did take place. The lifespan of the infected mice was about four days longer than that of the intact animals.

Thus, it was shown that fucoidan from *U. pinnatifida* suppresses the invasion of *P. falciparum* merozoites into erythrocytes both in vitro and in vivo, inhibiting the adhesion interaction between merozoites and erythrocytes through the binding of fucoidan sulfate to ligands on the cell surface of host cells, thereby inhibiting parasites. The authors recommended using a dose of fucoidan no higher than 100 mg/kg, as higher doses may have an anticoagulant effect [57]. To search for antimalarial compounds, red algae were also studied.

J. Marques et al. [53] presented the antimalarial and anticoagulant properties of sulfated galactan from the red alga *Botryocladia occidentalis*. The antimalarial activity of this compound is similar to that of heparin. In in vitro cultures, it significantly inhibited the growth of *P. falciparum*. The IC_50_ for galactan was 3.5 μg/mL, IC_90_—6.8 μg/mL (for comparison: for heparin, the IC_50_ was 4.1 μg/mL, IC_90_—8.0 μg/mL).

The authors did not establish a correlation between the molecular weight of the polysaccharide and growth inhibition of *P. falciparum* because the two best results were noted for galactan from the red alga *B. occidentalis* (Mw~700 kDa) and polysaccharide from the mollusc *L. grisea* (Mw~30 kDa). Compared with other SPS used by the authors in the experiment, galactan had the most pronounced anticoagulant properties. It has been suggested that the anticoagulant activity and antimalarial properties of galactan are not directly related.

Galactan actively suppressed the penetration of plasmodia into erythrocytes. Microscopic examination 20 h after treating erythrocytes with polysaccharides at a dose of 4 μg/mL showed an apparent decrease in the annular stages, i.e., delayed development of the parasite compared to samples untreated with galactan. Untreated control erythrocytes contained only trophozoites and schizonts. Under the influence of galactan, the rate of invasion of erythrocytes decreased (untreated—3.60 ± 0.10; those treated with galactan—1.10 ± 0.10); however, the maturation rate did not change dramatically (untreated erythrocytes—0.96 ± 0.06; erythrocytes treated with galactan—1.14 ± 0.08). Thus, the main mechanism of the antimalarial action of galactan is inhibition of the invasion of the parasite into erythrocytes [53].

It should be noted that free merozoites are present in the blood for a short period. This allowed the authors to assume that the binding process can also occur inside the erythrocyte. To confirm this position, heparin was added to the culture. Using confocal microscopy, it was shown that heparin penetrated living erythrocytes and bound intra-erythrocyte-developing merozoites [53].

In experiments on mice infected with *P. yoelii* and treated with intravenous galactan, this polysaccharide was shown to significantly reduce parasitemia compared to untreated animals. There were no side effects when the polysaccharide was administered to mice. Antibodies to *Plasmodium* antigens were found.

After 72 days, the mice were infected with plasmodia for the second time. All animals treated with galactan survived without any additional treatment and without disease symptoms until 42 days after the untreated secondary infection (follow-up period). On the positive side, galactan is not obtained from an animal object, i.e., in this case, there is no danger of prion contamination [53].

It is still impossible to talk about the biosynthesis of this promising compound because the mechanism of its enzymatic biosynthesis is unknown. However, an ample supply of algae in nature (found in abundance in Brazil) will make it possible to obtain sulfated galactan in sufficient quantities since its content in dry algae is about 50% [58].

SPS of red algae—galactans including carrageenans—are very promising antimalarial compounds. In [52], the authors investigated the antimalarial properties of a large panel of 50 compounds with inhibitory activity above 20%. Of these, 14 samples had high antimalarial activity at a concentration of 2 μg/mL. In this series, algal SPS were represented by highly sulfated carrageenans, lambda and iota. The authors believe that SPS inhibits the binding of merozoites to erythrocytes by disrupting the interaction of the receptor–ligand with sulfated receptors. It is believed that a variety of merozoite surface proteins mediate these initial contact effects through low-affinity interactions with the erythrocyte surface. Since many of these interactions are likely to be associated with surface sulfate receptors, the ability of heparin-like substances including those from algae to disrupt multiple interactions at several stages of invasion will probably be able to ensure the effectiveness of SPS for all strains of parasites and limit the emergence of drug resistance. This is especially true for SPS of seaweed.

The main mechanism of the pathogenic action of plasmodia is associated with a change in the properties of affected erythrocytes due to the formation of malarial tubercles (“knob structures”, “knobs”) on their surface, on the top of which there are special “handle” structures formed by the parasitic protein PfEMP1 (*P. falciparum* erythrocyte membrane protein 1). This protein plays the role of the main virulence factor. Moreover, the genome regions on which polypeptides exported by the parasite to the surface of erythrocytes are synthesized are highly polymorphic. The changes caused by exported proteins mediate the transformation of the physical characteristics of erythrocytes and impart adhesion properties to them [59]. Among the exported proteins, the PHIST family, containing 89 proteins, plays a significant role in enhancing the cytoadhesion of the reconstructed host cell [60]. The authors showed that sulfated polysaccharides can deactivate the proteins exported by the parasite, associated with control of the main virulent factor of the parasite *P. falciparum* erythrocyte membrane protein (PfEMP1) [61].

J.M. Mutisya et al. [62] devoted their work using the molecular docking method to the search for agents among SPS that inhibit the PHIST target. Using Sanger sequencing, 86 complete sequences were obtained among which 11 medicinal compounds with antiplasmodial activity were identified. In addition, ten compounds interacted with amino acid residues in the PHISTb (*P. helical* interspersed sub-telomeric b) and RESA (ring-infected erythrocyte surface antigen) domains, demonstrating potential activity against these proteins. At the same time, α-carrageenan from red alga interacted with both the reference and mutant proteins.

As potential sources of antimalarial agents, polysaccharides not only of different species of algae but also invertebrates can be used [63,64]. Thus, fucosylated chondroitin sulfate (FucCS), a unique highly sulfated GAG isolated from some marine organisms, was effective as an inhibitor of *P. falciparum* cytoadhesion to human lung endothelial cells and placental cryosection under static and flow conditions. This compound is low-toxic, rapidly destroyed the rosettes in a dose-dependent fashion, and blocked the development of various phenotypes of parasites, preventing the invasion of merozoites into erythrocytes. The authors proposed FucCS as a candidate for adjunctive therapy in patients with severe malaria. FucCS was also effective in preventing the parasite from adhering to the human placenta, eliminating it. Removal of the sulfated fucose branches from the compound practically eliminated the inhibitory effect, which indicates the central role of these branches in the inhibitory effect of the compound. The effect of FucCS appears to be similar to that of heparin, which inhibits invasion, adhesion, and rosette formation by binding to PfMP-1 regions and the surface protein of MSP-1 merozoites. FucCS can block the interaction of adhesion and invasion proteins with host receptors, which are glycoproteins constitutively expressed in erythrocytes, by binding to conserved regions of these proteins, such as the RII region [64].

FucCS treatment (1 mg/kg/animal per day) slowed down the death of C_57_Bl/6 mice infected with *Plasmodium berghei* ANKA (PbA) in an experimental model of cerebral malaria. The compound had a weaker anticoagulant and antithrombotic effect than heparin, and acted in vivo and in vitro at concentrations lower than those required to trigger its anticoagulant effect. Thus, the authors proposed FucCS as a promising candidate for the adjuvant therapy of severe malaria and the prevention of exacerbations of the disease [63,64].

The later work of M.F. Bastos et al. [65] presented materials on the study of antimalarial effect and the ability to inhibit cytoadhesion of heparan sulfate from the scallop *Nodipecten nodosus*. The polysaccharide is formed by repeating the disaccharide units of β-D-glucuronic acid 1 → 4 N-acetyl-α-D-glucosamine containing rare sulfate groups at the C2 or C3 positions of glucuronic acid. The polysaccharide blocked invasion up to 91% at the maximum dose (1000 μg/mL). It inhibited *P. falciparum* cytoadhesion to endothelial cells even at the lowest dose tested (1 μg/mL), and reached 86% and 100% inhibition of Pf-iEsCSA and Pf- iEsICAM-1, respectively, at the maximum dose (1000 μg/mL). The polysaccharide dose-dependently destroyed the rosettes at a rate similar to heparin, and showed antithrombotic activity without causing bleeding. Like the previous heparan sulfate, the authors recommended this compound for the adjunctive therapy of severe malaria.

A therapeutic composition consisting of chloroquine (100 nM) and fucoidan (20 μg/mL) has been proposed as a new agent for the prevention and treatment of malaria. The agent prevented the penetration of plasmodium into erythrocytes and did not give side effects, helping to overcome the resistance of the parasite to chloroquine [66].

Placental malaria is characterized by the cytoadhesion of infected erythrocytes and the expression of TNFα in the intervillous tissue, which can interfere with the transport of nutrients to the fetus and negatively affect its development. TNFα stimulates the cytoadhesion of erythrocytes in the capillaries of the placenta by enhancing the ligand bonds of *P. falciparum* Protein-1 (PfMP-1) with chondroitin sulfate A receptors.

The effectiveness of the combined use of SPS and drugs was also demonstrated in the work of Z. Rahmah et al. [67]. They investigated the effects of the seaweed extract of *Eucheuma cottonii* in combination with DHP (dihydroartemisinin-piperaquine) on the degree of parasitemia, cytoadhesion, TNFα level and fetal development in mice infected with *P. berghei*. DHP contains artemisin and chloroquine. *E. cottonii* is a red alga most abundant in Indonesia and containing SPS carrageenan in quantities ranging from 54% to 73%, depending on the place of growth and the type of algae.

The mice in the experiment were divided into four groups: (A)—intact, did not receive treatment with carrageenan; (B)—received DPH; (C)—animals were infected with *P. berghey* and given an extract; (D)—malaria-infected animals which received DHP and extract. Isolation of the placenta and fetus was performed on the 18th day after mating the mice.

Group A showed a high degree of parasitemia, TNFα expression, low fetal weight, and high cytoadhesion in the placenta.

In Group B, DHP therapy reduced parasitemia and cytoadhesion in the placenta. The same was true for the level of TNFα; however, the drug did not prevent low birth weight.

In Group C, there was a significant decrease in parasitemia and cytoadhesion in the placenta, a decrease in TNFα, and no decrease in birth weight.

In Group D, in comparison with group C, all indicators decreased even more. There was no low birth weight, i.e., the combination of the DHP drug with red algae extract containing a large amount of SPS carrageenan was experimentally effective against malaria, reducing cytoadhesion, parasitemia and the level of the proinflammatory cytokine TNFα and eliminating fetal developmental disorders.

Thus, SPS from marine hydrobionts (algae and marine invertebrates) can be a promising basis for creating antimalarial drugs that will have an effect both as independent treatments and as an addition to existing therapy for this protozoal infection. 

## 3. Leishmaniasis

Leishmaniasis is a transmissible disease of humans and animals. The disease occurs in the tropics and subtropics on all continents except Australia [4]. Every year, about one million new cases of infection are registered in the world, up to 75,000 people die from leishmaniasis, and about 400 million people are at risk of getting sick [5].

The disease is caused by 20 species of protozoa of the genus *Leishmania* transmitted by the bite of infected female mosquitoes of the genus *Phlebotomus* (more than 90 species) [68]. Leishmaniasis ranks third among vector-borne diseases after malaria and lymphatic filariasis [69].

In humans and other vertebrates (dogs, rodents, monkeys), leishmanias exist in an immobile stage, or amastigote, in the intestine of the carrier (as opposed to the flagellar stage, or promastigote. When mosquitoes bite infected mammals (a reservoir of about 70 species of animals, including humans) leishmanias enter the intestines of mosquitoes, where they begin to multiply. In the gastrointestinal tract of insects, amastigotes turn into promastigotes, which the female mosquito spits up at the site of the bite on the human skin, while anywhere from 100 to 100,000 amastigotes get into the wound. Here, they multiply in the cytoplasm of the cells of the reticuloendothelial system.

Neutrophils that appear at the entrance gate absorb parasites that do not multiply in these cells. During apoptosis, macrophages absorb neutrophils, where leishmanias turn within 2–5 days into an intracellular morphological form without flagellum, the amastigote. The breeding cycle of amastigotes is 24 h. In the phagolysosomes of macrophages, the parasite replicates and survives due to its resistance to the microbicidal mechanisms of these cells, NO and ROS [70]. The process begins in the skin at the point of penetration of the parasite in the form of a granuloma (leishmanioma).

There are three main forms of leishmaniasis: visceral (VL, black disease, kala-azar, causative agent *Leishmania donovani*), cutaneous (CL, pendinsky ulcer, causative agent *L. tropica*), and mucocutaneous (espundia, causative agent *L. brasiliensis*). In visceral leishmaniasis, the foci of infection are formed in the organs of the reticuloendothelial system [68].

The currently available list of antileishmanial drugs that affect various metabolic pathways of the parasite is limited, and the growing resistance to them is of concern. For the treatment of leishmaniasis, pentavalent antimony compounds, amphotericin B, lipid forms of amphotericin B, miltefosine, and azole preparations are mainly used. However, antimony, like other drugs, has a wide range of side effects and causes high mortality in HIV patients [71,72]. These circumstances have necessitated a search for more harmless means for the treatment and prevention of leishmaniasis.

As presented in the previous section on antimalarial agents, marine macroalgae and invertebrates contain compounds that can control certain pathogens. There is evidence that heparin-binding proteins (HBP) are present on the surface of leishmania and may play a significant role in the parasite’s life cycle, determining the success of the binding of the parasite to the host tissue. Thus, L.M.C. Cortes et al. [73] fractionated *L. braziliensis* promastigotes and isolated two heparin-binding proteins present on the promastigote surface. These proteins are involved in the adhesion of these parasites to Lulo cells (this insect cell line is a model for studying the interaction of insects and leishmania as it partially models adhesion events in the intestines of mosquitoes) or to a surface covered with heparin. About 860,000 HBP units are present on the surface of promastigotes. They inhibit the activity of protein kinase C (which will be discussed below) in the host organism by binding to heparin. In addition, the expression of HBP in *L. donovani* is associated with infectious forms of this parasite; HBP predominates in stationary-phase promastigotes, and successive culture passages of these parasites lead to a loss of the ability to bind to heparin.

Since SPSs have a molecular structure similar to heparin, they can bind various ligands, including protein receptors on the surface of the host cell [74].

**Figure 2:**
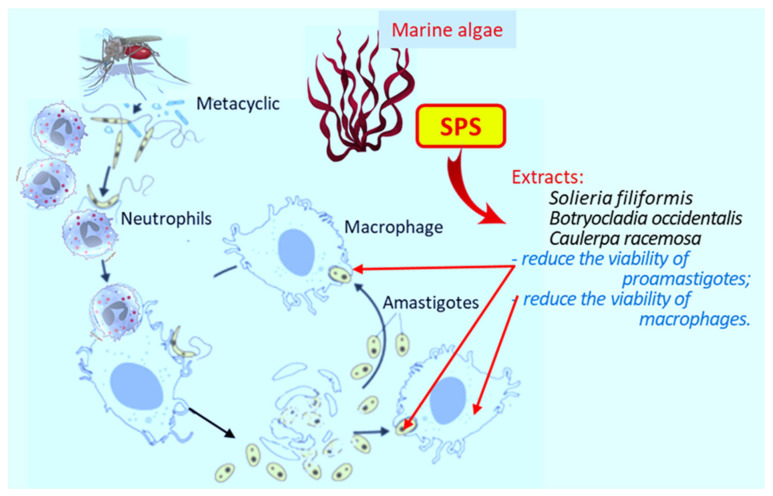
Highly purified SPS obtained from extracts of the red algae *Solieria filiformis*, *Botryocladia occidentalis*, and *Caulerpa racemosa* showed antileishmania activity.

Thus, to study this antileishmania effect, C.L. Pires et al. [75] used highly purified SPS obtained from extracts of the red algae *Solieria filiformis* (Sf), *Botryocladia occidentalis* (Bo), *Caulerpa racemosa* (Cr), and *Gracilaria caudata* (Gc). The authors evaluated their toxic effects and effects on the growth of *L. amazonensis* amastigotes (Figure 2).

The molecular weight of the polysaccharides was more than 200 kDa (Gc, Sf), 30 kDa (Bo), and 25 kDa (Cr), respectively.

Three of the four SPS were active against *L. amazonensis* (except for Gc), and they dose-dependently reduced the viability of amastigotes. The most active was extract Cr, which significantly reduced the viability of parasites (EC_50_—34.5 μg/mL). Polysaccharides derived from Bo and Sf showed moderate antileishmania activity, with EC_50_ of 63.7 μg/mL and 137.4 μg/mL, respectively. All polysaccharides reduced the survival of the J774 macrophage cell line, with CC_50_ values of 27.3 μg/mL, 49.3 μg/mL, 73.2 μg/mL and 99.8 μg/mL for Bo, Cr, Gc and Sf, respectively.

However, none of the polysaccharides decreased the viability of peritoneal macrophages. The authors suggested that the cytotoxicity of the compounds was responsible for the destruction of leishmania rather than any direct effect on the parasite. High molecular weight polysaccharides exhibited weak antileishmania activity. The authors proposed SPS as an alternative to heparin for the treatment of leishmaniasis, as they are more effective than alkaloids, terpenoids, and other natural algae products.

A more detailed study of the effectiveness of SPS as an antileishmanial agent is presented in the work of S.A. Minicante et al. [76]. The authors evaluated the effect of polysaccharides of seven species of algae (green, red, and brown) on cell cultures DH82, Vero, L929, MDCK, and U937. Polysaccharides in the studied concentrations (up to 160 μg/mL) did not have a toxic effect on cell cultures and showed an antileishmania effect against *L. infant* (except for two, *Agardhiella subulata* and *Hypnea cornuta*). In addition, a convincing result was obtained using SPS from *U. pinnatifida* and *Sargassum muticum*. At the maximum concentration of polysaccharides (160 μg/mL), the death rate of parasites was 100%, which was confirmed by morphological studies.

At an SPS concentration of 20 μg/mL, inhibition of the growth of leishmania and the presence of an abnormal and round shape of parasites took place. In cultures with a polysaccharide concentration of 80 μg/mL, leishmania cells were aggregated, rounded, and included individuals without flagella. In cultures with an SPS concentration of 160 μg/mL, it was impossible to find whole forms of protozoa, and only apoptotic bodies were determined. Noteworthy is the fact that SPS from *S. muticum* and *U. pinnatifida* at the same concentrations did not act on *Trypanosoma cruzi*, which indicates their specific action against leishmania.

Fucoidan was used orally three times a day for four weeks, starting 15 days post-challenge, at a dose of 200 mg/kg/day in a six-week experiment in BALB/c mice infected with both antimony-sensitive and resistant leishmania strains. The results obtained allowed Kar et al. to establish that this polysaccharide had an inhibitory effect on the multiplication of amastigotes of both strains in macrophages (inhibition > 93% at 50 μg/mL) and also eliminated the parasitic load in the liver and spleen of animals [77].

The participation of NO in the inhibition of intracellular reproduction of amastigotes by fucoidan was established. For this, an iNOS inhibitor (2-amino-5,6-dihydro-6-methyl-4H-1,3-thiazine (AMT)) was used. The in vitro inhibitory effect of fucoidan was markedly reduced after 24 h in the presence of AMT (86% and 77% reductions for AG83 and GE1F8R, respectively). Fucoidan had no direct effect on the viability of promastigotes at doses of 25 and 50 μg/mL. However, slight inhibition (9% and 7% for AG83 and GE1F8R, respectively) was found when the polysaccharide was exposed to a dose of 100 μg/mL for 48 h. The viability of mouse peritoneal macrophages remained unchanged up to 150 μg/mL fucoidan.

In vivo experiments on mice intravenously infected with *L. donovani* promastigotes of strains AG83 and GE1F8R investigated the parasitic load in the liver and spleen. After six weeks all animals treated with fucoidan remained healthy and none of the groups showed a decrease in body weight. The action of fucoidan was dose-dependent and changed over time.

At a lower dose (25 μg/mL), the percentage of parasite death varied between antimony-sensitive (62%) and resistant (45%) strains. However, at a 50 μg/mL concentration for 24 h, inhibition was almost complete (96% and 93% for AG83 and GE1F8R, respectively).

To clarify whether fucoidan provides long-term protection, the cured mice were re-infected two months after the initial infection. The parasite load progressed rapidly in the placebo-treated mice, while the fucoidan-treated mice treated with the polysaccharide at a dose of 200 mg/kg/day were resistant to reinfection, which was observed up to 7 weeks; i.e., fucoidan therapy can form acquired immunity against both susceptible and resistant strains of leishmania. However, the authors emphasized that the mouse model does not reproduce all the features of active human visceral leishmaniasis.

Visceral leishmaniasis is accompanied by impaired proliferation of T-lymphocytes. In this regard, S. Kar et al. [77] conducted a study of the effect of fucoidan on this process. Four weeks after fucoidan treatment, mice infected with AG83 and GE1F8R showed a 12.8- and 11.2-fold increase in spleen T cell proliferation. A comparison of the cytokine expression profile was performed, and it was shown that in both groups of infected animals (AG83 and GE1F8R) the IFNγ, IL-12, and TNFα indices increased after four weeks in AG83 mice, by 7.5, 6.4 and 5.8 times, respectively, and in GE1F8R animals by 6.5, 5.4 and 5.8 times, respectively. IL-10 and TGFβ decreased in AG83 mice treated with fucoidan by 79% and 75%, respectively, and in GE1F8R mice treated with polysaccharide by 72% and 70%, respectively.

Thus, in in vivo situations, fucoidan can provide protection against both antimony-sensitive and resistant strains by direct action on the induction of NO and ROS, stimulation of splenocyte proliferation, and switching the balance of cytokines from the TH2 to the TH1 regime, which creates host protection against leishmania [77].

Leishmanias survive in a hostile environment in macrophages by inhibiting their defense mechanisms, namely, the formation of nitric oxide (NO) and the production of reactive oxygen species (ROS). Parasites use strategies to interfere with a wide range of signalling processes in macrophages, including protein kinase C (PKC), the JAK2/STAT1 cascade, and the MAP kinase pathway [78]. PKC signalling activates NF-kB, a universal transcription factor involved in expressing protective molecules [79,80]. Lipophosphoglycan on the cell surface of *Leishmania* promastigotes is a potent inhibitor of PKC activity in vitro in intact macrophages.

Depletion of PKC creates favourable conditions for the proliferation of *L. donovani* and promotes the survival of the parasite in host cells. At the same time, the expression of atypical isoforms (PKCε and PKCζ) increases, while Ca^2+^-dependent PKC-β decreases in patients with leishmaniasis. Deactivation of PKCβ correlates with increased IL-10 production and disease progression. Furthermore, PKC signal transduction activates NF-kB, a universal transcription factor involved in expressing protective molecules [79,80]. In addition, there is evidence that *Leishmania* themselves express phosphatases targeting host cell molecules, which also contributes to the intracellular survival of the parasite [81].

Earlier [77], it was found that oral administration of fucoidan in mice at a dose of 200 mg/kg/day for three weeks infected with antimony-sensitive *L. donovani* strain resulted in the relieving of parasitic load in the liver and the inhibition of >93% of amastigotes in macrophages. As mentioned earlier, this healing effect is associated with a Th2 to Th1 switch. In addition, splenocytes from mice treated with fucoidan produced higher levels of NO and ROS. 

G. Sharma et al. [70] investigated the cellular mechanisms underlying the antileishmanial effect of fucoidan in macrophage culture in vitro using a model of visceral leishmaniasis. A commercial preparation of fucoidan from *Fucus vesiculosus* contained 97% fucose and trace amounts of galactose, xylose and uronic acid.

When using monoclonal antibodies against IFNγ, TNFα and IL-12, a significant decrease in the protective effect of fucoidan was observed (parasite suppression was 57%, 52% and 63%, respectively) compared to mice receiving fucoidan (parasite inhibition was 99%).

In another series of experiments, mice were infected and then treated with fucoidan and AMT (2-amino-5,6-dihydro-6-methyl-4H-1,3-thiazine). This compound is a known inhibitor of the enzyme NO synthase (NOS) [82]. Mice treated with AMT had a weaker reduction in parasitic load than those treated with fucoidan. The mRNA levels of iNOS (the main enzyme that promotes NO formation) four weeks after infection were increased in infected mice treated with fucoidan (induction 9.15-fold), and remained at the same level after six weeks. Fucoidan mediated the induction of iNOS and proinflammatory cytokines dependent on the activation of p38 and ERK1/2 MAPK, which ultimately led to the activation of NF-kB and the associated antileishmanial response.

Treatment of infected macrophages with fucoidan induced a time-dependent increase in PKC-a, -b1 and b-2 kinase activities. The maximum PKC-a activity was 2.1 times higher 60 min after treatment, and the PKC-b1 and b2 activity were 2.7 and 2.67 times higher than in the untreated control, respectively.

Thus, fucoidan not only protects against leishmanias, it also activates the host’s immune response. The results obtained by the reviewed authors indicate the effectiveness of this polysaccharide as a powerful immunoregulator for inclusion in the treatment of visceral leishmaniasis, including when caused by antimony-resistant leishmanias.

As a new therapeutic method for leishmaniasis, M.H.M. Hoseini et al. [83] suggested using chitin. To prove this, the authors injected female BALB/c mice subcutaneously with *Leishmania* promastigotes and microparticles of chitin and/or chitosan for two weeks. The animals were sacrificed 12 weeks later, except for one group two weeks after infection. In the groups receiving chitin (0.6 ± 0.12 mm) and chitosan (1.2 ± 0.8 mm), the mean lesion sizes were significantly smaller than in the control group (6.2 ± 1.7 mm).

The parasite load in the lymph nodes of the polysaccharide-treated mice was considerably lower than in the lymph nodes of the control animals. Chitin microparticles induced cell proliferation and increased the production of TNFα and IL-10, that is, they acted as immunomodulators. Moreover, chitin was more effective in experimental leishmaniasis than chitosan. On the other hand, Riezk et al. [84] showed that chitosan and its derivatives at pH 6.5 were approximately 7–20 times more active than at pH 7.5, both against extracellular promastigotes and against intracellular amastigotes of *L. major* and *L. mexicana*. In this case, chitosan with a high molecular weight was more active. This pattern stimulated the production of nitric oxide and reactive oxygen species in both infected and non-infected leishmania macrophages in a time and dose-dependent manner. The rhodamine-labelled polysaccharide was absorbed by pinocytosis and accumulated in the parasitophorous vacuole of macrophages infected with leishmania. Thus, chitosan and chitin require further research as antileishmania drugs.

## 4. Trypanosomiasis

Trypanosomiasis is a group of transmissible protozoal diseases caused by flagellate protozoa of the genus *Trypanosoma*. The World Health Organization lists these diseases as one of the six most dangerous tropical diseases, distinguishing between American (Chagas disease) and African trypanosomiasis.

There are three stages in the developmental cycle of trypanosomes: (i) the invasive stage, in which the tripomastigote has an elongated shape and the flagellum is an undulating membrane and is therefore very mobile; (ii) the epimastigote exists inside of the carrier; and (iii) the immobile amastigote exists as an intracellular parasite of vertebrates.

The causative agent of American trypanosomiasis, *Trypanosoma cruzi*, is spread by the bite of a triatomine bug and is endemic to the continental region of Latin America. However, in recent years the disease has been found in the United States, Canada, and many European countries [85]. To date, about 6–7 million people around the world are sick with trypanosomiasis and the population at risk of infection is about 65 million.

The invasive stage for the vector, as well as for a vertebrate animal, are metacyclic trypomastigotes, which insects receive when feeding on animals and humans. The faeces of these insects carry the trypomastigotes into wounds or scratches on the skin and cause infection [86]. Moreover, they are also able to invade any germline host cells [86].

The main sites of parasitic trypanosomes are the smooth muscles of the heart and gastrointestinal tract. The life cycle ends when uninfected triatomaceous bugs feed on the blood of infected mammalian hosts and ingest parasites, which grow and differentiate in the insect’s gastrointestinal tract and eventually migrate to the hindgut. 

The disease can be transmitted through insect vectors, through the placenta from mother to fetus, through blood and tissue transplants, through ingestion of parasitized meat or freshly squeezed fruit juice, or as a result of intra-laboratory infection.

The causative agents of African trypanosomiasis (African sleeping sickness) are *Tripanosoma brucei gambiense* and *Tripanosoma brucei rhodesiense*; the carrier is the tsetse fly. The insect receives metacyclic trypomastigotes from humans or animals (pigs, antelopes, cattle) which act as the host for this parasite. The most anthropophilic insect species is *G. palpalis*. In 2019, 992 cases of African trypanosomiasis were reported. The population at risk of infection is about 65 million.

The minimum invasive dose of trypanosomes is 300–400 parasites. A fly gives off about 400,000 trypanosomes in one bite. One bite of an infected fly is enough for a person to fall ill with sleeping sickness. From about ten days after the bite, a person becomes a source of infestation.

Once inside the host, trypomastigotes migrate and spread throughout the body through the circulatory and lymphatic systems, after which they begin to multiply by binary fission. Ultimately, they disrupt the blood–brain barrier, which leads to infection of the central nervous system and the death of almost 100% of untreated patients [87].

Specific therapy is based on using nifurtimox and benznidazole, both of which are nitroheterocyclic agents that induce the formation of reactive oxygen species. However, these drugs have low efficacy and adverse side effects (impaired liver function, pancreas, skin irritation) [88]. In addition, the annual global cost of treating infected patients is approximately USD 24.7 billion, with 10% of this in the United States and Canada [89]. In connection with this situation, the search for inexpensive, effective and low-toxic compounds that can be used against trypanosomes continues.

As such agents, D. Leal et al. [90] used SPS from brown algae *Lessonia* spp. In addition to alginic acid, brown algae contain sulfated polysaccharides consisting mainly of L-fucopyranose residues linked by α1 → 3 and α1 → 4 glycosidic bonds. It has already been said above that sulfated fucoidans are of great interest due to their multifaceted high biological activity.

From the extract of *Lessonia* spp. blades, fucoidan was isolated and structurally characterized, then further investigated for trypanocidal activity against epimastigotes, a non-infectious replicative form found in the intestine of insect vectors.

The IC_50_ of fucoidan for epimastigous *T. cruzi* was 250 ± 3.92 μg/mL. The activity of fucoidan was lower than that of the drug nifurtimox (28.84 ± 0.84 μg/mL). However, the polysaccharide showed low toxicity in mammalian cells, with a selectivity index of 1.47 towards the *T. cruzi* parasite. S. Kar et al. [77] reported that commercial fucoidan significantly increased ROS and NO levels in *L. donovani*-infected macrophages. The authors showed that fucoidan’s possible mechanism of action in this case is associated with an increase in ROS, causing oxidative stress in parasites which lack endogenous antioxidant systems and are therefore susceptible to oxidative stress.

At the same time, if the polysaccharide has antioxidant activity [91] and partially reduces the concentration of free radicals, this can lead to a decrease in the trypanocidal activity of the SPS. 

To expand the possibilities for practical application of SPS, W.M. Souza et al. [92] used a sulfated polysaccharide as raw material for the preparation of an anti-trypanosome nanopreparation. To increase SPS activity, the authors synthesized a preparation containing silver nanoparticles and fucoidan obtained from the brown alga *Spatoglossum schroederi*.

These algae synthesize three types of bioactive heterofucans, A, B, and C, of which A has the highest content of fucoidan in comparison with others. Furthermore, it was previously shown in animal studies that the studied polysaccharides were not genotoxic or mutagenic either in vitro or in vivo [93,94].

W.M. Souza et al. [92] obtained seven fractions rich in sulfated fucoidans from the algae extract, which were analyzed by electrophoresis in agarose gel after staining with toluidine blue. Only one band was detected in fractions FO, 5v, and FO6v, which indicated the purity of the obtained polysaccharides. Afterwards, the fucoidans were used to obtain nanoparticles based on silver (Ag). The darkening of the solution marked the completion of the nanoparticle formation process.

**Figure 3:**
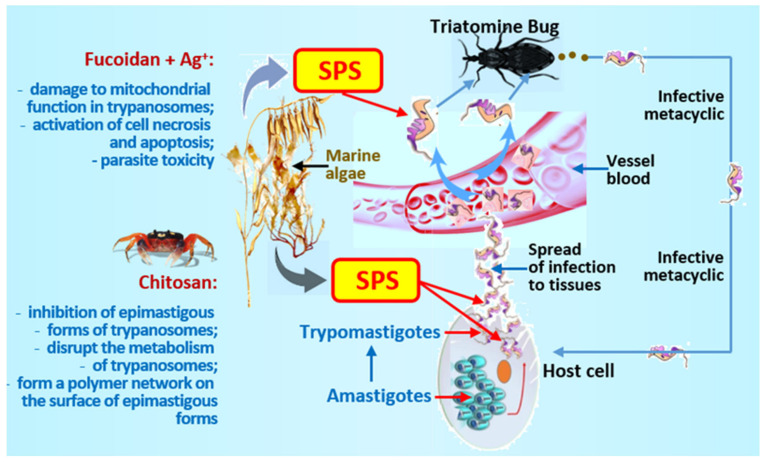
Sulfated biopolymers from marine hydrobiont fucoidans (brown algae) in combination with silver nanoparticles, as well as chitosan from crustaceans, have an antitrypanosomal effect.

The study of the antiparasitic activity of AgFuc, Ag and the FO, 5v fraction showed that at the lowest concentration (25 μg) all of the samples were inactive. Silver had the lowest toxicity against the parasite. FO, 5v affected the epimastigote form of trypanosomes only at the highest concentration used (100 μg). Moreover, its influence was more pronounced after 48 h (59.4 ± 1.4%). The highest time-dependent effect was obtained with AgFuc nanoparticles (after 24 h, the inhibition rate was 58.9 ± 1.2%; after 48 h, 67.3 ± 2.1%). Sample FO, 5v did not kill the parasites. Instead, AgFuc significantly increased the number of necrotic and apoptotic cells (Figure 3).

Rhodamine is a marker of mitochondrial function. Application of FO, 5v reduced the number of rhodamine-stained parasites. AgFuc caused damage to the function of mitochondria in parasites, resulting in their death. According to the authors, parasites may have receptors that recognize polysaccharides, particularly fucoidans, facilitating the penetration of nanoparticles into the parasite’s cytoplasm. Inside the parasite, they decompose and the silver component causes the formation of ROS, damaging mitochondria and causing the death of the parasite. These results are very interesting and promising for creating antitrypanosomal drugs; however, they require further in-depth studies.

Cell labelling with annexin V-FITC and P1 helps differentiate apoptotic, necrotic and viable cells. AgFuc significantly increased the number of cells stained with P1, indicating a higher level of necrosis than control samples. There were also many cells stained with annexin (19.7%), which indicated the apoptotic effect of the nanopreparation.

Another promising study concerns the use of chitosan oligosaccharides [95]. We present data on chitosan among the results obtained using SPS since the mechanisms of antiparasitic action of these compounds are similar.

The commercial preparation of chitin is obtained from the shells of marine arthropods. Every year in the world, seafood processing generates from 6 to 8 million tons of shell waste of which, most is thrown away [96]. Chitosan is a copolymer of N-acetyl-D-glucosamine (GlcNAc) and D-glucosamine (GlcN) obtained by partial deacetylation of chitin. Chitosan is biocompatible, nontoxic and biodegradable, and is therefore widely used in many fields [97]. In addition, the cationic nature of chitosan leads to enhanced bioadhesion on the membranes of microorganisms and fungi [98].

DaSilva et al. [95] used this property of chitosan against trypanosomes. However, its high molecular weight, degree of deacetylation, and, most importantly, poor solubility remains major obstacles to its widespread use in medicine. Therefore, the authors obtained small soluble chitooligosaccharides (COS) by enzymatic hydrolysis of chitosan using chitosanase from *B. toyonensis*.

Under the microscope, trypanosomes looked like elongated cells with a single thin flagellum and a smooth surface. After 72 h of incubation with COS, a network was observed on the surface of epimastigote forms of *T. cruzi* which adhered to the parasites, forming aggregates. The use of energy-dispersive X-ray spectroscopy (EDS) made it possible to establish that the grid consisted of organic material. The authors were unable to determine whether the network was COS or an extravasate of the internal contents of the parasites. Still, it appeared to be a combination of an oligomer and an extravasate. Thus, studies have shown that COS can form a polymer network that interacts with the parasite, preventing movement or possibly the exchange of nutrients by the parasite through membrane receptors by disrupting its metabolism. The oligomers also caused a time-dependent inhibition of the epimastigote forms of trypanosomes.

Thus, COS can be used to control *T. cruzi*, possibly in combination with drugs or in drug delivery systems, to enhance their action or overcome drug resistance.

## 5. Schistosomiasis

Schistosomes, or blood flukes, are the causative agents of schistosomiasis. In humans, the three species are most often found are *Shistosoma haematobium*, the causative agent of genitourinary schistosomiasis, and *S. japonicum* and *S. mansoni*, the causative agents of intestinal schistosomiasis [68]. It is estimated that at least 236.6 million people required treatment in 2019, of whom only 105.4 million received treatment. Helminthiasis occurs primarily in tropical and subtropical developing countries [99].

The main pathological manifestations of Japanese schistosomiasis are granuloma and subsequent liver fibrosis caused by the retention of worm eggs in this organ, which leads to fatal irreversible damage in the liver and intestines of the host [100,101]. At the same time, a dominant CD4 + Th2 immune response mediated by IL-4 and IL-13 develops, leading to the development of granulomas and fibrosis. During the development of liver fibrosis, macrophages of the M2 phenotype act as a key cell population (30%). These cells play a critical role in the immune response, as they contribute to the TH2 response and are required to suppress the Th1 response.

It is known that fucoidans have anti-inflammatory effects, inhibiting LPS-induced inflammation in macrophages and blocking the signalling pathway of the nuclear factor NF-kB, TLR4 [102]. A commercial preparation of fucoidan from the brown alga *Sargassum hemiphyllum* reduced the expression of cytokines (TIMP-1, CXCL-1, MCP-1, and MIP-2), reducing the intensity of the development of lung fibrosis in mice [103].

X. Bai et al. [104] used fucoidan obtained from the brown alga *F. vesiculosus* as an anti-inflammatory and hepatoprotective agent in experimental schistosomiasis (500 mg/kg twice a day for 40 days). When using fucoidan to treat mice infected with schistosomes, a significant decrease in the size of liver granulomas and the degree of fibrosis was observed during the infectious process. The levels of mRNA and anti-inflammatory cytokines (IL-4 and IL-13) increased, as did infiltration of the liver and spleen tissue with Treg cells as well as the levels of IL-10 and TGFβ mRNA.

Among the splenocytes stimulated in vitro by fucoidan, the number of Treg cells increased along with increased expression of chemokine receptors CCR4 and CXCR5 on Treg-cells. In addition, an increase in the levels of IL-4 and IL-13 mRNA was observed in macrophages.

Thus, the use of fucoidan in schistosomiasis reduces pathological changes in the liver and prevents the progression of the process caused by *S. japonica*, which may be a new potential strategy for the treatment of patients with pathology caused by this helminthiasis in the future.

## 6. Cryptosporidiosis

Cryptosporidiosis is a parasitic disease of the intestinal tract of mammals, including humans, caused by the protozoa *Cryptosporidium parvum*. *Cryptosporidia* are capable of multiplying not only in cells but also extracellularly [105]. Cryptosporidiosis is spread by food, often through contaminated water. The agent causes severe watery diarrhea in humans and other mammals [106], and is especially dangerous for immunocompromised individuals. Cryptosporidiosis is a major problem for animal farming, as these protozoa are one of the most common enteropathogens in young farm animals and poultry, resulting in large economic losses [107]. In Europe, about 4.7 million disease cases are registered annually, with about 500 deaths per year [108].

The only drug approved by the Food and Drug Administration (FDA, Silver Spring, MD, USA) for the treatment of immunocompromised people with cryptosporidiosis is nitazoxanide, the effectiveness of which is low; therefore, as with other parasitic diseases, a search is underway for effective new drugs for the treatment and prevention of this disease.

Infection with cryptosporidia occurs when the host animal swallows the oocysts of the pathogen, after which they secrete sporozoites as they pass through the stomach and duodenum. Sporozoites attach to intestinal epithelial cells, envelop the host cell membrane, and develop into trophozoites.

Sporozoites, however, cannot directly interact with intestinal epithelial cells. Instead, they contain a glycocalyx, a filamentous layer of branched carbohydrates [109]. This acts as a protective barrier [110] and includes a high level of transmembrane mucin glycoproteins of the proteoglycan type [111]. Proteoglycans are macromolecules located on the cell surface or in the extracellular matrix in which one or more glycosamine chains are covalently linked to a membrane or secreted protein. Various pathogens use these structures to invade host cells [112,113,114].

A. Inomata et al. [115] suggested that *C. parvum* interacts with GAG on host cells, and some polysaccharides can inhibit the parasite’s attachment to cells [116]. The authors evaluated the anticryptosporidial effects of five sulfated polysaccharides on parasite invasion and found that they all have an inhibitory effect on this process. In this case, the most powerful effect was exerted by heparin at a dose of 1 μg/mL. Among the studied SPS was fucoidan, which had a dose-dependent inhibitory effect, reducing the invasion of parasites by about 50% at a concentration of 100 μg/mL.

The results of this study also indicated that polysaccharides (in particular, this was shown in the fucoidan model) compete with some factor(s) on the surface of the parasite and that this is are involved in the invasion of HCT-8 cells. It has also been shown that cell surface heparan sulfate plays a role in *C. parvum* invasion in vitro.

The authors did not aim to study the antiparasitic action of fucoidan in detail. However, they showed the mechanism of action of SPS when interacting with parasites and cells, as well as the need to continue research in this direction in order to create new effective drugs against cryptosporidiosis.

H. Maruyama et al. [116] investigated the effect of native (obtained from the sporophylls of *U. pinnatifida*) and desulfated fucoidans on the adhesion of cryptosporidia to the cell culture of human intestinal epithelium (Intestinal 407), as well as the infectious process in newborn mice. The adhesion of *C. parvum* to intestinal cells was significantly reduced when the cells were treated with a low dose of fucoidan (1%, 50 μg/mL). The number of *C. parvum* oocysts in mice receiving fucoidan decreased by almost five times compared with control animals that did not receive the polysaccharide. Thus, the authors have convincingly proven that fucoidan effectively inhibits the growth of *C. parvum* in mice and also prevents the adhesion of parasites to the epithelial cells of the human intestine. At the same time, fucoidan can inhibit cryptosporidium due to direct binding to functional mediators derived from *C. parvum* in the intestinal epithelial cells of newborn mice. However, the desulfated fucoidan had little effect on the growth of parasites.

Chitosan has promising potential in the prevention and treatment of cryptosporidiosis. To assess the effect of two chitosans (Chitosan NAG and Chitosan Mix) on oocyst secretion in newborn CD-1 mice, *C. parvum* oocysts were orally inoculated, treated with chitosans and compared with those of untreated animals [117]. Paromomycin, a classic drug used in veterinary medicine, was used as a control. Treatment with both chitosans and paromomycin significantly reduced the release of parasites in infected animals that received chitosan (−56%, −34.5%, 58%, respectively).

In the in vitro experiments, after 24 h incubation at 37 °C a significant decrease in the viability of cryptosporidium oocysts was observed (>95%). In addition, paromomycin, chitosan NAG, and chitosan Mix all dose-dependently inhibited the reproduction of *C. parvum* in the NCT-8 and Caco-2 cell lines. The authors considered these results as the first evidence of the effectiveness of chitosan as an anticryptosporidial compound.

## 7. Trichomoniasis

*Trichomonas vaginalis* is a flagellated protozoan that causes trichomoniasis, a sexually transmitted disease in humans. Infection is possible through non-sterile gynecological instruments and gloves. The parasite lives on the surface of the epithelium of the urogenital tract of women and men. The parasite replicates by binary fission of the nucleus. The trophozoite form is the only stage of this protozoan [118].

The disease is widespread. According to the WHO, about 170 million people are infected with vaginal trichomonas. In developed countries, the prevalence of this pathology is 2–10%; in developing countries, it reaches 40%. 5-nitroimidazole is used to treat the disease [119]. Oral administration of this medication remains the recommended treatment regimen for trichomoniasis. However, the adverse side effects on the gastrointestinal tract, allergenic profile, and increasing drug resistance of parasites necessitate a search for new ways to combat trichomonas [120].

As with other parasitic diseases, scientists are searching for new effective compounds in order to create drugs against this infection, including among biologically active substances from marine hydrobionts, particularly algae. Thus, C.B.S. Telles et al. [121] analyzed the activity of five heterofucans from the brown alga *S. filipendula*, which have strong antiproliferative and antioxidant effects. Two out of the five polysaccharides were inactive towards *Trichomonas trophozoites*

Three fucans showed activity after 24 h of vaginal treatment in mice. The polysaccharides exerted a cytotoxic effect on protozoa, almost completely suppressing their viability. As shown in this work, the degree of sulfation of fucans is an essential factor in anti-*T. vaginalis* activity. The authors placed great hopes on algal polysaccharides; however, they also drew attention to the fact that further serious research is needed in order to clarify the complete structure of these polysaccharides, the configuration of glycosidic bonds, their position, and the number of sulfate groups and branch points.

## 8. Conclusions

As follows from the above materials, SPS from marine hydrobionts are promising antiparasitic compounds. Parasites’ development of resistance to traditional drugs leads to the loss of the effectiveness of existing drugs as therapy. In addition, the toxicity and adverse side effects of many drugs used to treat parasitic infections stimulates the search for alternative remedies. Active screening of biologically active natural compounds, including among the metabolites of marine aquatic organisms on which basis effective drugs can be obtained, has led to the development of new strategies in the therapy and prevention of parasitic diseases.

SPS from marine algae and invertebrates are practically nontoxic, and only in rare cases slightly toxic in the body. Polysaccharides such as, for example, fucoidan, have long been widely used by the population of many countries and are produced in the form of food additives [17,18].

However, the mechanisms of the antiparasitic action of these unique compounds at the cellular and molecular levels are far from being fully understood. It can be considered that at present there is an accumulation of knowledge, and attempts are being made to explain many processes, as the interaction of each type of parasite with different SPS is specific and each requires a special approach.

Numerous studies have shown that both algae extracts and the metabolites of these aquatic organisms, including SPS, have not only powerful antiparasitic, but also antioxidant, anti-inflammatory, immunomodulatory and antitoxic potential [18,122,123], which cannot but enhance their action against parasitic invasion. Therefore, it is necessary to study polysaccharides with a characterized structure in order to determine how such important structural parameters as monosaccharide composition, type of glycoside bond, molecular weight, the content of sulfate groups, and uronic acids affect the antiparasitic activity of SPS. Such materials have already been obtained when studying the effectiveness of SPS in bacterial and viral infections [124] and, to a lesser extent, in parasitic invasions [53,57,125]. This also seems to occur in the interaction of SPE with parasites of various taxonomic groups [53,57]. Thus, it is necessary to expand research on compounds with a high antiparasitic effect in order to determine the relationship between their structure and biological activity.

Of great interest is the study of the nature of the direct action of SPS on protozoa using contemporary methods, as is the case in the study of the interaction between bacteria and polysaccharides. These compounds bind to the surface of the bacteria, causing damage to the membrane or leakage of nutrients from the microorganism. This is confirmed by the discovery of nucleic acids [126] and proteins [127] released after the treatment of bacteria with SPS. It is possible to carry out the same research with unicellular and multicellular parasites. It is also possible that SPS traps and binds nutrients in the environment, leading to a loss of bioavailable of nutrients for parasites and causing a decrease in viability or even death in protozoa.

A careful approach in determining the antiparasitic action of SPS requires an adequate extrapolation of the data [128] obtained in vitro to the field of application in vivo, given that most studies of marine polysaccharides at present are conducted outside the body. The possibility of a discrepancy between the concentrations of drugs used for in vitro experiments to affecting the body’s cells and protozoans and the actual conditions of the human body should be borne in mind.

Questions about the targets for the SPS of marine hydrobionts on each species of protozoa, as well as about whether parasites can develop resistance to these compounds, also require in-depth study.

Despite the abundance of unresolved issues, SPS from marine hydrobionts, which combine high antiparasitic potential with antitoxic, anti-inflammatory, immunomodulatory and antioxidant properties, are a promising basis for creating new drugs, food supplements, and functional food products to fight parasitic infections. It is also necessary to conduct in-depth studies of the possibility of the combined use of antiparasitic drugs and SPS to reduce the toxicity of antiparasitic drugs and eliminate their undesirable side effects. The multivalent action of SPS has been realized at the cellular and molecular level. It is a significant factor in increasing the antiparasitic therapeutic potential of biologically active substances in aquatic organisms. SPS, unlike many drugs, has a substantial number of targets for the implementation of their action, and their antiparasitic effect is a combination of several possible mechanisms leading to the death of parasites.

Difficulties in the development of drugs based on SPS remain, largely due to the complexity of their standardization as they need to be standardized in terms of such physicochemical parameters as molecular weight, monosaccharide composition and degree of sulfation, and structure of side chains as well as the type or combination of types of bonds between fucose and other residual monosaccharides that make up the SPS. Obtaining chemically pure, structurally characterized and homogeneous samples with low molecular weight or oligomeric fractions with polydispersity indices close to unity from native polysaccharides is a difficult task. One of the approaches to solving it consists in the use of polysaccharide-degrading enzymes such as fucoidan hydrolases (fucoidanase), alginate lyase, and carrageenans sulfatases [129,130].

Despite this, the combination of new methods including genetic modification of pathogens, molecular docking, bioimaging, and the use of physicochemical methods for research, which led to the standardization of high-throughput screening platforms in drug development, will undoubtedly allow these strategies to be applied to the creation of antiparasitic drugs based on biologically active substances from marine hydrobionts.
